# Multiple Roles for VEGF in Non-Melanoma Skin Cancer: Angiogenesis and Beyond

**DOI:** 10.1155/2012/483439

**Published:** 2012-10-17

**Authors:** Kelly E. Johnson, Traci A. Wilgus

**Affiliations:** Department of Pathology and Integrated Biomedical Science Graduate Program, The Ohio State University, Columbus, OH 43210, USA

## Abstract

Vascular endothelial growth factor (VEGF) is known to play a critical role in the development of non-melanoma skin cancers. VEGF is a potent pro-angiogenic factor and it is elevated in mouse and human skin tumors. The use of transgenic and knockout mice has shown that VEGF is essential for tumor development in multiple models of skin carcinogenesis and, until recently, the mechanism of action has been primarily attributed to the induction of angiogenesis. However, additional roles for VEGF have now been discovered. Keratinocytes can respond directly to VEGF, which could influence skin carcinogenesis by altering proliferation, survival, and stemness. *In vivo* studies have shown that loss of epidermal VEGFR-1 or neuropillin-1 inhibits carcinogenesis, indicating that VEGF can directly affect tumor cells. Additionally, VEGF has been shown to promote tumor growth by recruiting macrophages to skin tumors, which likely occurs through VEGFR-1. Overall, these new studies show that VEGF carries out functions beyond its well-established effects on angiogenesis and highlight the need to consider these alternative activities when developing new treatments for non-melanoma skin cancer.

## 1. Introduction

Non-melanoma skin cancer (NMSC) is the most commonly diagnosed type of cancer. Over 2 million patients are treated for these cancers each year in the USA alone [1], resulting in nearly $1.5 billion total direct costs annually [2]. Unlike many other types of cancer, the rates of NMSC continue to rise [3], indicating the need to increase research and identify new, more effective therapies. NMSCs are primarily caused by chronic exposure to ultraviolet (UV) light from the sun, although chemical exposure, chronic wounds, and viral infection can be risk factors as well [1, 4]. There are two main types of NMSC: basal cell carcinoma (BCC) and squamous cell carcinoma (SCC). BCCs account for about 80% of skin cancers [3] and although these tumors are rarely metastatic, patients have a high risk of developing additional tumors within 5 years of diagnosis [5]. SCCs make up roughly 16% of all skin cancers [3] and are typically more aggressive than BCCs, posing a higher risk for metastasis and leading to approximately 2,500 deaths annually [1]. The risk of developing skin cancer is very high in the general population, as one in five people will develop skin cancer in their lifetimes [6]; however, certain populations such as transplant patients are at an even greater risk [7, 8]. 

**Figure 1:**
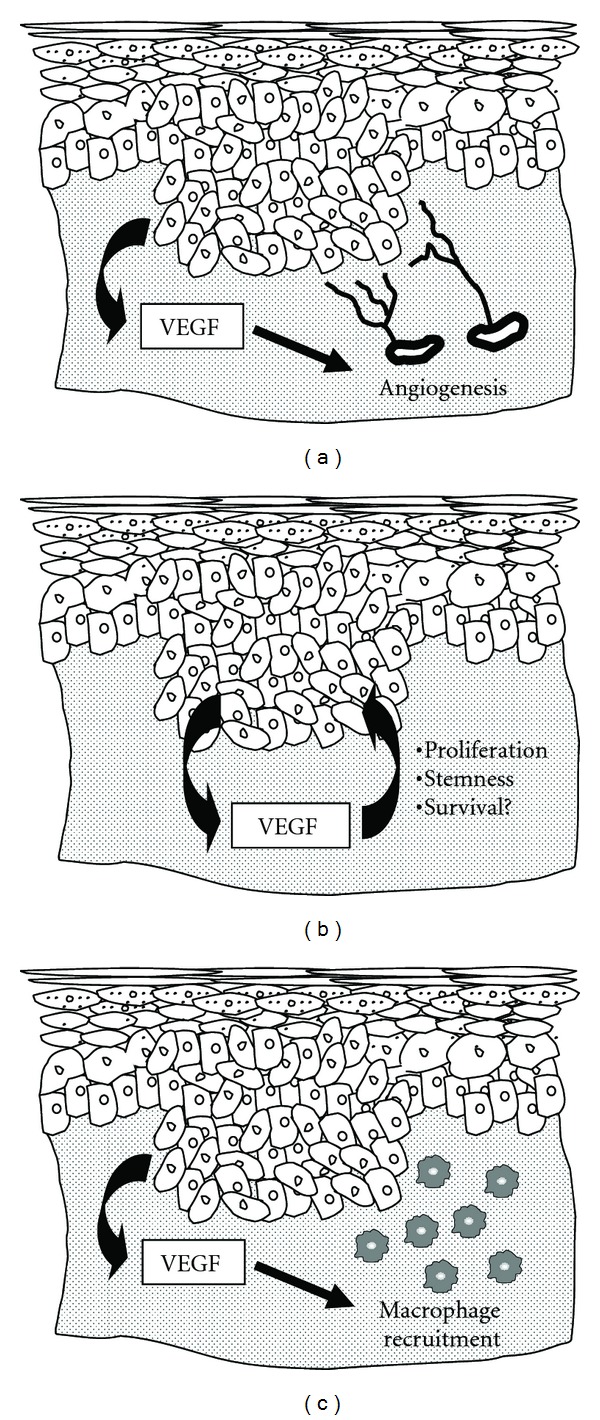
Tumor-promoting actions of VEGF in skin carcinogenesis. Epithelial tumor cells produce large amounts of VEGF in the skin, as depicted by the arrow on the left side of each panel. Traditionally, VEGF has been recognized only for its ability to stimulate angiogenesis through paracrine actions on endothelial cells (a). However, additional functions of VEGF have now been described. Recent studies have suggested that VEGF can affect epithelial cells in an autocrine manner by stimulating proliferation, maintaining stemness, or possibly by promoting survival (b). Additionally, macrophages can be recruited to skin tumors by VEGF through paracrine mechanisms. These macrophages are capable of producing an array of mediators that can support the growth of tumor cells in the skin.

Angiogenesis, the growth and expansion of the vasculature, is an important process in the growth and metastasis of many cancers, including NMSC [9]. Vascular endothelial growth factor (VEGF) is a potent pro-angiogenic factor and several studies have established a critical role for VEGF in skin cancer [10]. VEGF transgenic and conditional knockout mice subjected to skin carcinogenesis protocols, such as the well-established two-stage chemical carcinogenesis model [11, 12], have demonstrated that VEGF promotes skin carcinogenesis through the induction of angiogenesis [13, 14]. Additionally, several recent studies have now uncovered direct effects of VEGF on keratinocytes and skin tumor cells. These studies have suggested that in addition to enhancing angiogenesis, VEGF may promote skin carcinogenesis by altering the survival, proliferation, or stemness of keratinocytes and tumor cells in an autocrine manner [15–18]. Furthermore, immune cells such as macrophages can respond to directly VEGF [19, 20] and recent studies indicate that VEGF recruits macrophages to skin tumors [21]. This review will highlight our current knowledge of the angiogenic and newly discovered non-angiogenic activities of VEGF that contribute to non-melanoma skin cancer, which are summarized in Figure 1.

## 2. Angiogenesis and VEGF 

 Angiogenesis is a key process in the growth and spread of many cancers, including skin cancer. Typically, angiogenesis is required for tumors to grow beyond 1-2 mm in size and offers a route for tumor cells to disseminate to secondary sites [22]. Because of this, tumor angiogenesis has been an attractive and promising therapeutic target [23]. To induce angiogenesis, tumor cells and cells within the tumor microenvironment must alter the balance of pro- and anti-angiogenic factors, favoring an “angiogenic switch” [24]. When pro-angiogenic signals outweigh anti-angiogenic signals, it allows for capillary sprouting through the proliferation and migration of endothelial cells. Eventually, the newly formed vessels supply the tumor with oxygen and nutrients required for continued growth. Many pro-angiogenic factors have been identified and characterized, including basic fibroblast growth factor (bFGF), interleukin-8 (IL-8), platelet-derived growth factor (PDGF), placental growth factor (PlGF), transforming growth factor-*β* (TGF-*β*), and vascular endothelial growth factor (VEGF). 

VEGF-A (referred to as VEGF throughout this article) is a 45 kDa heterodimeric heparin-binding protein belonging to the family of vascular endothelial growth factors. At least 5 splice variants of VEGF have been identified in humans, including VEGF_121_, VEGF_145_, VEGF_165_, VEGF_189_, and VEGF_206_ [25, 26]. VEGF binds to three known receptors: VEGF receptor-1 (VEGFR-1), VEGF receptor-2 (VEGFR-2), and neuropilin-1 (NRP-1) [27–29]. VEGFR-1 and VEGFR-2 are tyrosine kinase receptors characterized by a seven immunoglobulin-like extracellular domain, a single transmembrane region, and an intracellular tyrosine kinase domain [30]. NRP-1 is a single pass transmembrane protein that binds semaphorins as well as some isoforms of VEGF [31]. NRP-1 functions as a coreceptor for the VEGFRs, enhancing their activity [32]; however, NRP-1 may be able to signal independently of VEGFRs in response to VEGF, particularly in tumor cells [33]. VEGF is well characterized as a potent inducer of angiogenesis and functions as a survival factor and mitogen for endothelial cells [34, 35]. In general, VEGF is expressed at low levels by epidermal keratinocytes and is upregulated during many pathological processes such as wound healing, psoriasis, and skin carcinogenesis [36–38]. VEGF production by keratinocytes can be induced by many stimuli including hypoxia, transforming growth factor-*α*, keratinocyte growth factor, UV radiation, and the tumor promoter 12-O-tetradecanoylphorbol-13 acetate (TPA), while VEGF production is inhibited by the transcription factor Fra-1 [39–46]. 

## 3. VEGF and Angiogenesis in Skin Tumors

Strong evidence has demonstrated that VEGF plays an important role in skin carcinogenesis. In human skin, VEGF is expressed at low levels in normal epidermis, with more differentiated epidermal cell layers generally expressing more VEGF than less differentiated epidermal cells [47–49]. Several studies have confirmed that VEGF levels are elevated in tumor cells compared to normal epidermal cells using immunohistochemistry and *in situ *hybridization techniques [47–49]. Tumor cells of human BCCs tend to show weak VEGF expression [47, 48, 50] with positive tumor cells predominantly localized to the invading margin [50]. In contrast, SCCs, which are typically more aggressive than BCCs, display more intense and widespread staining, with higher expression in tumor cells localized near infiltrating inflammatory cells [47, 50]. Furthermore, VEGF expression is elevated in poorly differentiated SCCs compared to well differentiated tumors [50]. Vessel density is also high in SCCs, especially in late-stage SCCs, compared to normal skin, actinic keratoses, BCCs, or early-stage SCCs [48, 49].

In mice, acute exposure to tumor promoters such as TPA or UV light causes upregulation of VEGF and induction of angiogenesis in the skin [38, 51–53]. VEGF expression patterns in murine models of skin carcinogenesis mimic what is observed in human tumors. VEGF is low in murine skin and increases stepwise during tumorigenesis [38]. A functional role for VEGF in skin tumor angiogenesis has been demonstrated through the use of transgenic and conditional knockout mice. Both K6-VEGF and K14-VEGF transgenic mice which overexpress VEGF in epidermal keratinocytes show elevated blood vessel density in the skin and in skin tumors compared to controls [13, 14, 54]. VEGF transgenic mice are also more susceptible to two-step chemical carcinogenesis [13, 14]. In addition to containing a larger number of blood and lymphatic vessels both within and surrounding skin tumors, K14-VEGF mice develop chemically-induced tumors more rapidly and also have a dramatically higher incidence of metastasis than controls [14]. Conversely, conditional K14-VEGF knockout mice have reduced blood vessel density in tumors and are much more resistant to chemical carcinogenesis [55]. 

VEGF also plays a role in UV-induced skin carcinogenesis. In addition to inducing papillomas and SCCs, UV exposure increases VEGF levels and neovascularization in the skin [52, 53, 56]. Inhibition of VEGF in the skin with compounds such as epigallocatechin-3-gallate (ECGC) and myricetin leads to a decrease in angiogenesis and a reduction in the number of UV-induced skin tumors [56–58]. 

Evidence from orthotopic skin tumor models has also shown a link between VEGF, angiogenesis, and tumor development [59, 60]. SCC-13 cells transfected with VEGF form invasive, highly vascularized tumors when injected subcutaneously or intradermally into nude mice [59]. Similarly, tumors arising from a malignant HaCaT cell line, which produce large amounts of VEGF, initiate angiogenesis more quickly and to a larger degree than HaCaT cell lines which form benign tumors [60]. Furthermore, treatment with VEGFR-2 blocking antibodies reduces endothelial cell proliferation and vessel density in tumors derived from the malignant cell lines to levels comparable to benign cell lines. In addition, VEGFR-2 antibody treatments reduce tumor growth and invasiveness, suggesting that VEGF promotes tumor growth by inducing angiogenesis. Taken together, the evidence from human tumors and animal models demonstrate that VEGF is critical for the development, growth, and spread of skin tumors, and these findings have been largely attributed to the promotion of angiogenesis by VEGF. 

## 4. Autocrine Roles for VEGF in Skin Carcinogenesis

Although dermal cells such as macrophages, fibroblasts, and other cell types are known to produce VEGF, epidermal keratinocytes are believed to be the principle source of VEGF in the skin [36, 45, 55, 61]. In addition to stimulating angiogenesis through its actions on endothelial cells, recent evidence has demonstrated that VEGF can have direct effects on keratinocytes. Several groups have now identified VEGF receptors on keratinocytes, suggesting the possibility of autocrine VEGF signaling. Currently, there is some disparity in the exact receptor profiles that have been described. Some studies have identified VEGFR-1, VEGFR-2, and NRP-1 on keratinocytes [18, 62]; however, others do not detect VEGFR-2 [15–17]. Our lab has shown that VEGF induces the proliferation of cultured primary human keratinocytes through VEGFR-1 [15] and this finding has been confirmed by others in murine keratinocytes [17]. VEGF has also been shown to induce the migration of primary keratinocytes *in vitro *[63]. Additionally, VEGFR-1 is expressed in mouse and human skin tumor cells and in squamous cell carcinoma cell lines [17], suggesting that VEGF could affect tumor cells directly.

Autocrine functions for VEGF in keratinocytes and skin tumor cells have also been suggested by recent functional studies performed* in vivo* [16, 17]. Lichtenberger et al. utilized various conditional knockout mice to uncover a direct role of VEGF in skin carcinogenesis using the K5-SOS model, in which the ras activator Son of Sevenless is constitutively activated in the epidermis [17]. In this model, K5-SOS mice develop skin tumors spontaneously and tumors can be induced rapidly by wounding the skin [17, 64]. Keratinocytes were shown to overexpress VEGF in the K5-SOS model, and K5-specific deletion of VEGF reduced tumor development in these mice. Loss of keratinocyte VEGF also lead to a decrease in vessel density and a decrease in tumor cell proliferation, and VEGF was able to enhance keratinocyte proliferation *in vitro*. Because VEGFR-1 expression was detected in murine and human skin cells, epidermal VEGFR-1 was deleted in K5-SOS mice. A reduction in papilloma development and tumor cell proliferation was observed in conditional VEGFR-1 knockout mice compared to controls, while blood vessel density was unaffected. VEGFR-1 knockdown in SCC tumor cell lines was also shown to slow proliferation. Together, these studies establish a direct role for VEGF in skin carcinogenesis, wherein VEGF stimulates tumor cell proliferation through VEGFR-1.

Interestingly, an autocrine loop between VEGF and NRP-1 has also been discovered. Using the two-stage chemical skin carcinogenesis model, Beck et al. recently reported an effect of VEGF on CD34^+^ cancer stem cells (CSCs) [16]. CD34^+^ tumor cells were shown to express higher levels of VEGF than CD34^−^ tumor cells or normal keratinocytes [16]. Epidermal overexpression of VEGF increased the pool of CD34^+^ CSCs, while inhibition of VEGFR-2 activity with DC101 or conditional deletion of VEGF in the epidermis reduced the CSC pool and diminished CSC proliferation, in addition to reducing the number of established tumors. Interestingly, VEGF-overexpressing CSCs were found to have high levels of NRP-1. Conditional deletion of NRP-1 completely blocked tumor formation in the chemical carcinogenesis model compared to control mice which all developed papillomas. In addition, when conditional NRP-1 knockout mice were crossed with VEGF transgenic mice, VEGF was unable to promote tumor growth, even though efficient tumor angiogenesis was still observed. Overall, the results suggest that epithelial cell-derived VEGF regulates CSCs in an autocrine manner. 

In addition to affecting epithelial cell proliferation and stemness, a recent study suggested that VEGF may also directly affect keratinocyte survival *in vitro*.Zhu et al. showed that exposure to UV light, the primary causative agent of NMSC, increased the expression of VEGFR-1, VEGFR-2, and NRP-1 in primary normal human keratinocytes *in vitro *and in human epidermis *in vivo *[18]. VEGFR upregulation was found to be a result of UV-induced oxidative stress. UV exposure also resulted in activation of VEGFR-1 and VEGFR-2. Interestingly, VEGF was able to protect keratinocytes from apoptosis following exposure to moderate (300 J/m^2^) but not high (700 J/m^2^) doses of UV. Activation of VEGFR-2, but not VEGFR-1, was responsible for the observed increase in keratinocyte survival. Although these results will need to be confirmed *in vivo*, they suggest that VEGF could function as a survival factor for keratinocytes following UV exposure. 

## 5. Paracrine Roles for VEGF in Skin Carcinogenesis

In addition to endothelial cells, some immune cells also express VEGF receptors, supporting the idea that VEGF can have paracrine effects that are not related to its pro-angiogenic activity. For example, monocytes and macrophages express VEGFR-1 and VEGF has been shown to be a chemoattractant for these cells [19, 20]. Tumor-associated macrophages, particularly M2 macrophages, are believed to promote tumor growth and invasion and well as angiogenesis [65, 66]. Recently, Linde et al. used an orthotopic tumor model in which control or VEGF-transfected HaCaT cells were injected subcutaneously into mice [21]. VEGF-driven HaCaT tumors were larger, more vascular, more invasive, and had higher numbers of infiltrating M2 macrophages compared to control tumors. Depletion of macrophages reversed the effects of VEGF overexpression, indicating that VEGF was influencing tumor development by affecting macrophages. In this model, VEGF stimulated the recruitment of macrophages to the tumors but was not sufficient to polarize them. Additional tumor- and macrophage-derived IL-4 and IL-10 were responsible for M2 polarization. These studies indicate that in addition to promoting angiogenesis, VEGF can influence skin carcinogenesis by recruiting immune cells.

## 6. Conclusions

Strong evidence has established a critical role for VEGF in the development of non-melanoma skin cancers. VEGF is produced by the skin in response to tumor-promoting agents such as TPA and UV light, and skin tumors are known to express elevated levels of VEGF. In mouse studies, VEGF increases angiogenesis and tumor growth, while the loss of VEGF inhibits skin carcinogenesis. To date, these findings have been primarily attributed to the potent pro-angiogenic effects of VEGF. However, the presence of VEGF receptors on non-endothelial cell types, such as keratinocytes and macrophages, has expanded our view of the potential functions of VEGF. Indeed, new evidence suggests that VEGF can impact skin carcinogenesis by directly affecting keratinocytes, tumor cells, and immune cells. While there is no doubt that VEGF plays an important role in skin carcinogenesis, more work is required to characterize the various mechanisms by which VEGF contributes to this process and to understand the relative importance of each of these pathways. Further studies will have to be carried out to determine whether these newly described alternative functions of VEGF can be targeted to treat NMSC.
